# Anti-CGRP monoclonal antibodies in chronic migraine with medication overuse: real-life effectiveness and predictors of response at 6 months

**DOI:** 10.1186/s10194-021-01328-1

**Published:** 2021-10-07

**Authors:** Edoardo Caronna, Victor José Gallardo, Alicia Alpuente, Marta Torres-Ferrus, Patricia Pozo-Rosich

**Affiliations:** 1 Neurology Department, Hospital Universitari Vall d’Hebron, Department of Medicine, Universitat Autònoma de Barcelona, Barcelona, Spain; 2 Headache and Neurological Pain Research Group, Vall d’Hebron Research Institute, Department of Medicine, Universitat Autònoma de Barcelona, Barcelona, Spain

**Keywords:** Migraine, Medication overuse, CGRP, Monoclonal antibodies, Erenumab, Galcanezumab

## Abstract

**Background:**

In daily practice, anti-CGRP monoclonal antibodies (MAbs) may be useful in chronic migraine (CM) with medication overuse (MO), but data is limited. We evaluated their effectiveness in a real-life clinical cohort.

**Methods:**

This is a prospective study conducted in CM patients with and without medication overuse treated with monthly MAbs during 6 months (erenumab/galcanezumab). We collected headache characteristics, including acute medication intake, through an electronic diary. We compared patients (1) with and without MO at baseline, (2) with and without ongoing MO after treatment, defining MO resolution as < 10 or 15 days/month of acute medication intake, according to analgesic type, during the 6-month treatment.

**Results:**

Of 139 CM patients completing 6-month treatment with anti-CGRP MAbs, 71.2% (99/139) had MO at baseline. After 6 months, patients with and without MO at baseline had significant and similar proportions of ≥50% reduction in migraine days/month (MO: 63.6% vs. non-MO: 57.5%, *p* = 0.500). 60.6% (60/99) no longer satisfied MO definition. Reduction in headache frequency compared to baseline occurred in both MO-ongoing and MO-resolution group, although those who stopped overusing had a greater improvement (headache days/month: − 13.4 ± 7.6 vs. -7.8 ± 7.2, *p* < 0.0001). No differences in MO resolution were observed according to the MAbs used. Baseline lower pain severity was associated with MO resolution (OR [95%]:0.236[0.054–0.975]; *p* = 0.049).

**Conclusions:**

In real-life anti-CGRP MAbs are as effective in CM patients with MO as in patients without it and facilitate MO cessation. Reduction in headache frequency and acute medication days/month occurs regardless of whether patients stop overusing or not.

**Supplementary Information:**

The online version contains supplementary material available at 10.1186/s10194-021-01328-1.

## Introduction

Medication overuse (MO) is defined as an acute medication intake of more than 10 or 15 days/month, according to the type of drug used, being present in more than 50% of chronic migraine (CM) patients [1]. At present, withdrawal of overused medication with concomitant initiation of preventive treatment is the best recommended strategy for MO [2]. In this context, the availability of anti-calcitonin gene-related peptide (CGRP) monoclonal antibodies (MAbs) [3], a new class of migraine preventive treatment, represents a promising way to facilitate the cessation of MO; however, real-life effectiveness needs to be proven, as data is scarce [4–7].

Clinical trials with anti-CGRP MAbs included a small proportion of patients who were overusing acute medication, although they were not designed to evaluate this population. However, post-hoc subgroup analyses have observed efficacy in reducing headache frequency and acute medication intake [8–10] in those overusing.

Thus, we decided to analyze in a real-life clinical cohort the effectiveness of anti-CGRP MAbs after 6 months of treatment in patients with CM with and without MO, focusing on predictors of response.

## Methods

This is a prospective study performed in a Spanish specialized Headache Clinic. From February 2019 to April 2021, we included consecutive patients who started treatment with anti-CGRP MAbs, following the criteria required by the Spanish government for anti-CGRP MAbs prescription (more than 8 migraine days, with failure of at least 3 migraine preventive medications, being onabotulinumtoxinA (BTX-A) one of them for CM) and in line with the European Headache Federation guideline on anti-CGRP MAbs use [11].

At the initial visit, diagnoses of CM and presence of MO were done using International Classification of Headache Disorders – 3 criteria [12]. Patients completed an electronic headache diary (eDiary), starting 1 month prior to the first dose of anti-CGRP MAbs and throughout all treatment period to monitor headache frequency (migraine days/month-MDM; headache days/month-HDM) and severity. Acute medication (AM) intake was collected as acute medication days/month (AMDM) and number of pills/month (AM burden).

Anti-CGRP MAbs were administered monthly (Erenumab 140 mg or Galcanezumab 120 mg + 240 mg for the loading dose). Patients were not educated to stop acute medication and no detoxification protocol was done prior to anti-CGRP MAbs treatment. No changes were made in other concomitant preventive medications, being the doses stable for at least 3 months for oral preventive treatments and 6 months (2 cycles) in the case of onabotulinumtoxinA. Treatment response was evaluated after 24 weeks in a follow-up visit, when eDiary and baseline variables were reassessed. For the analysis of pain severity, which was collected daily, a mean pain intensity of all monthly headache days was calculated. We defined MO resolution if patients presented < 15 days/month of simple analgesics/non-steroidal anti-inflammatory drugs (NSAIDs) or < 10 days/month of triptan intake during the treatment period (24 weeks).

### Statistical analysis

We reported nominal (categorical) variables as frequencies (percentages) while continuous variables as mean ± standard deviation when normally distributed or median and interquartile range (IQR) when non-normally distributed. We checked normality assumption of quantitative variables through visual methods (Q-Q plots). We assessed statistical significance comparisons between MO resolution group (MO-R) and MO-ongoing group (MO-O) by Pearson’s chi-square when comparing categorical variables or Fisher’s exact test. Finally, independent t-test was used for continuous variables with normal distribution and Mann-Whitney U test for the rest.

For our preplanned multivariate analysis, a both (forward and backward) stepwise logistic regression was used as an exploratory analysis to identify clinical factors associated with MO response. Included variables were either based on statistical significance from bivariate tests (*p* ≤ 0.01) or considered clinically relevant (age and gender). The final set of features (predictors) was chosen according to their effect in minimizing (optimizing) the Akaike Information Criteria (AIC) of the resulting model. Classification model was computed in 60% of the initial sample (randomly selected as a train subset) and tested in the rest of data (40% as a test subset). A 10-fold cross-validation (10-fold CV) was also performed in order to validate the prediction outcomes of our logistic regression classifier, assessing the model performance by their accuracy, kappa value and the area under the curve (AUC) in both subsets (train and test).

We did not conduct a statistical power calculation prior to the study because the sample size was based on the available data. No missing values were obtained. No adjustments for multiple comparisons were made to the statistical inferences, but exact *P* values were reported to allow post adjustments. We considered two-tailed test *P* values < 0.05 statistically significant. All analyses were done using R Core Team, 2021.

## Results

Since February 2019, 316 patients had started anti-CGRP MAb as a prophylactic treatment for migraine (59 episodic migraine; 257 CM). At the time of the analysis, 44.0% (139/316) were CM patients who had completed 6 months of treatment (69.1% Erenumab and 30.9% Galcanezumab). After 6 months, 11.5% (16/139) of CM patients had discontinued treatment (13 because of lack of efficacy, 2 had lack of tolerability and 1 pregnancy desire). In regards to safety, 30.9% (43/139) reported mild adverse events, mainly constipation (76.7%, 33/43).

From this cohort of 139 CM patients, 81.3% were females, mean age was 47.0 ± 9.5 years old and 71.2% (99/139) had MO (NSAIDs overuse: 38.4% (38/99); triptan overuse: 79.8% (79/99). There were no differences in the proportions of MAbs (Erenumab vs. Galcanezumab) used between patients with and without MO. Other baseline characteristics and main differences between the two groups are shown in Supplementary Table 1.

**Fig. 1:**
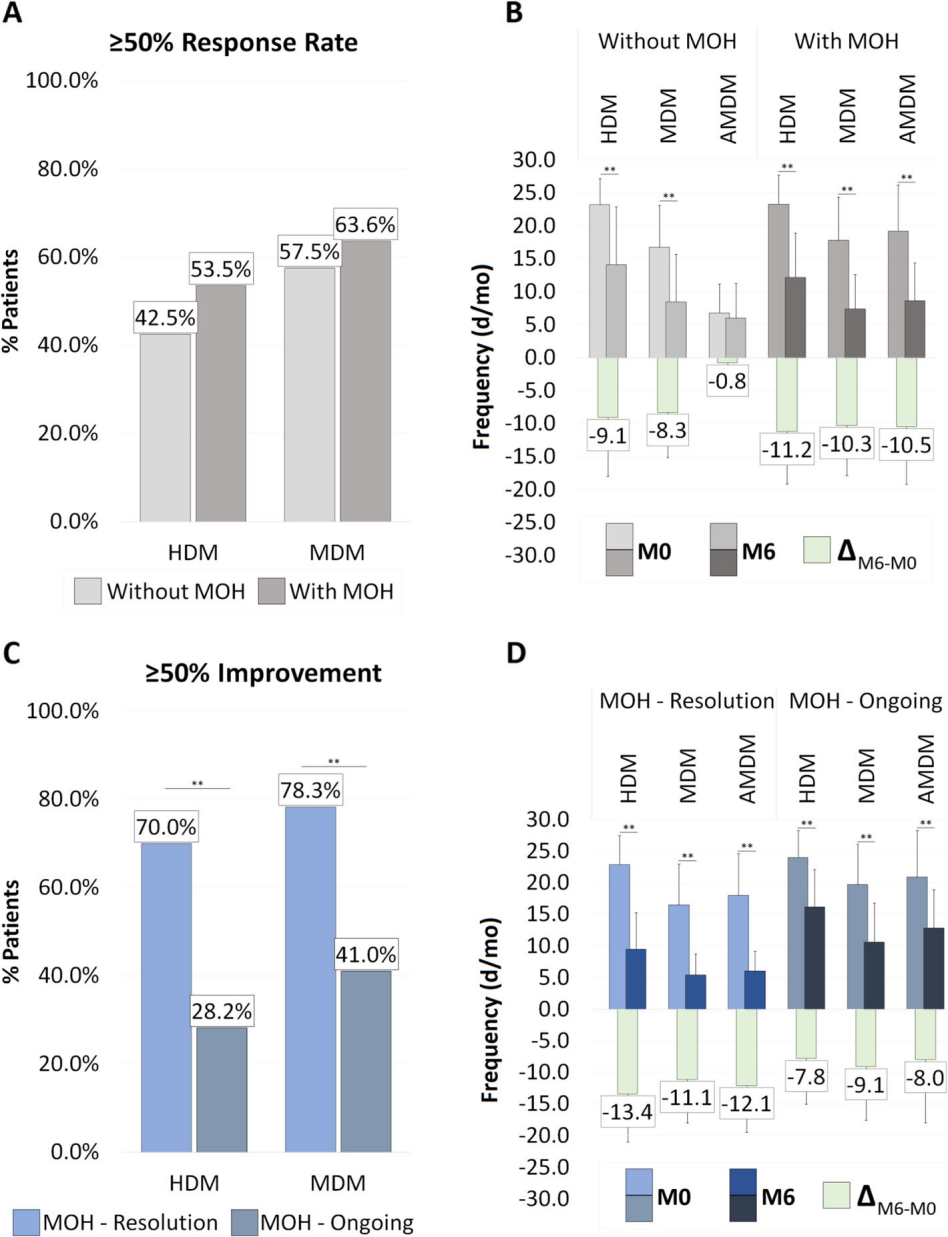
Anti-CGRP MAbs ≥50% RR (**A; C**) and mean change in primary outcomes after 6 months of treatment (**B; D**) in study patients. In section A, we report ≥50% RR in HDM and MDM in CM patients with MO (light gray, 99/139) and without MO (dark gray, 40/139) at baseline. In section C, we also report ≥50% RR in patients with basal MO (99/139), comparing them according to their MO response after 6 months of treatment: MO resolution (light blue, 60/99) vs. MO ongoing (dark blue, 39/99). In section B, we report the mean change in HDM, MDM and AMDM after 6 months of treatment in CM patients with MO (light gray, 99/139) and without MO (dark gray, 40/139) at baseline. In section D, we also report the mean change in HDM, MDM and AMDM in patients with basal MO (99/139), comparing them according to their MO response after 6 months of treatment: MO resolution (light blue, 60/99) vs. MO ongoing (dark blue, 39/99). In green is shown the mean difference between Month 0 and Month 6 (Δ). ^**^ *P*-value < 0.01 (AB: Significance assessed with Fisher’s exact test; CD: Significance assessed with paired t-test). RR: Response Rate; MO: medication overuse; d/mo: days per month; p/mo: pills per month; HDM: headache days/month; MDM: migraine days/month; AMDM: acute medications days/month

After 6 months, 50.4% of patients (70/139) achieved a ≥ 50% reduction in HDM while 61.9% (86/139) in MDM. No statistically significant differences in HDM or MDM response rates were found according to baseline presence of MO (Supplementary Table 1 and Fig. 1A). In regards to MO, 60.6% of patients (60/99) no longer satisfied MO definition (MO-R) while 39.4% (39/99) kept overusing acute medication (MO-O).

### Medication overuse subanalysis

We conducted a subanalysis comparing patients with and without MO resolution at 6 months (Table 1). Differences in HDM, MDM and AM between the two groups are shown in Fig. 1B. Concerning baseline variables, patients with ongoing MO presented higher frequency of MDM (MO-R: 16.5 ± 6.5 vs. MO-O: 19.7 ± 6.4 d/mo; *p* = 0.020), higher score in a 0–3 pain severity scale (MO-R: 1.60 ± 0.46 vs. MO-O: 1.85 ± 0.45; *p* = 0.020), higher analgesics intake (MO-R: 18.0 ± 6.6 vs. MO-O: 20.9 ± 7.4 d/mo; *p* = 0.049) and benzodiazepine use (MO-R: 1.2 ± 0.9 vs. MO-O: 5.2 ± 2.1 d/mo; *p* = 0.034). The MO-O group had more anxiety in the Beck Anxiety Inventory scale (BAI) (MO-R: 15.5[19.0] vs. MO-O: 22.0[20.0]; *p* = 0.020). We also observed greater proportions of previous failure to onabotulinumtoxinA in the MO-O group (MO-R: 33.3% vs. MO-O: 53.8%; *p* = 0.048). There were no differences between groups in regards to the type of MAbs used.

**Table.1:** Baseline characteristics and their association with medication overuse resolution after 6 administrations of anti-CGRP MAbs

	***MO Resolution*** *(n = 60)*	***MO Ongoing*** *(n = 39)*	*P value*
***Demographics***			
Age, mean (SD), y	46.7 (9.5)	49.2 (8.2)	0.172^**§**^
Gender (female), n (%)	48 (80.0)	33 (84.6)	0.561^**†**^
***Baseline disease characteristics***			
Duration of migraine disease, mean (SD), y	24.9 (13.9)	28.8 (11.9)	0.146^§^
Chronification time, mean (SD), y	11.8 (7.8)	13.2 (7.3)	0.359^§^
Aura, n (%)	12 (20.0)	11 (28.3)	0.345^†^
Allodynia, n (%)	29 (48.3)	20 (51.3)	0.774^†^
Unilateral pain side, n (%)	41 (68.3)	34 (87.2)	0.053^†^
Pain quality, n (%)			
Oppressive	28 (46.7)	20 (51.3)	0.653^†^
Throbbing	41 (68.3)	26 (66.7)	0.862^†^
HDM, mean (SD), d/mo	22.9 (6.0)	24.0 (5.4)	0.347^§^
**MDM, mean (SD), d/mo**	16.5 (6.5)	19.7 (6.4)	**0.020**^**§**^
**Headache pain intensity, mean (SD), 0–3 score**	1.60 (0.46)	1.85 (0.45)	**0.011**^**§**^
***Preventive treatment***			
Anti-CGRP Treatment, n (%)			0.416^†^
Erenumab 140 mg	40 (66.7)	29 (74.4)	
Galcanezumab 120 mg (loading dose 240 mg)	24 (33.3)	9 (25.6)	
Prior preventive classes failures, n (%)			0.876^†^
3 Classes	5 (8.3)	3 (7.7)	
4 Classes	24 (40.0)	15 (38.5)	
≥ 5 Classes	31 (51.7)	21 (53.8)	
Prior BTX-A efficacy^*^, n (%)			**0.048**^**†**^
**Partial**	36/54 (66.7)	18/39 (46.2)	
**Failure**	18/54 (33.3)	21/39 (53.8)	
Concomitant preventive treatment, n (%)	36 (60.0)	29 (74.4)	0.142^†^
Oral concomitant medication, n (%)	31 (51.7)	26 (66.7)	0.140^†^
BTX-A concomitant medication, n (%)	20 (33.3)	18 (46.2)	0.200^†^
***Basal acute medication***			
MO NSAIDs, n (%)	23 (38.3)	15 (38.5)	0.990^†^
MO Triptans, n (%)	46 (76.7)	33 (84.6)	0.336^†^
**Acute medication frequency, mean (SD), d/mo**	18.0 (6.6)	20.9 (7.4)	**0.049**^**§**^
NSAIDs frequency, mean (SD), d/mo	8.2 (7.9)	10.5 (9.5)	0.421^§^
Triptans frequency, mean (SD), d/mo	12.1 (7.3)	14.2 (8.3)	0.194^§^
**BZD frequency, mean (SD), d/mo**	1.2 (0.9)	5.2 (2.1)	**0.034**^**§**^
**Acute medication burden, mean (SD), p/mo**	24.2 (9.4)	31.2 (13.6)	**0.006**^**§**^
***Basal disease impact, disability and burden***			
Disability (MIDAS), median [IQR]	71.5 [71.0]	95.0 [65.0]	0.117^‡^
Headache-related impact (HIT-6), mean (SD)	67.7 (12.3)	69.5 (18.8)	0.561^§^
**Anxiety (BAI), median [IQR]**	15.5 [19.0]	22.0 [20.0]	**0.020**^**‡**^
Depression (BDI-II), median [IQR]	11.0 [15.5]	12.0 [25.5]	0.226^‡^
***Treatment response rate***			
**HDM 50% RR, n (%)**	42 (70.0)	11 (28.2)	**< 0.0001‡**
**MDM 50% RR, n (%)**	47 (78.3)	16 (41.0)	**< 0.0001**^**‡**^

**Bold** font indicates statistically significant variables

^*^93/99 patients were previously treated with BTX-A

^§^Significance assessed with independent *t*-test

^†^Significance assessed with Fisher’s exact test or linear trend chi-square test (preventive classes failures)

^‡^Significance assessed with Mann-Whitney U test

*MO* Medication overuse, *SD* Standard deviation, *IQR* Interquartile range, *y* Years, *d/mo* Days per month, *p/mo* Pills per month, *HDM* Headache days/month, *MDM* Migraine days/month, *BTX-A* OnabotulinumtoxinA, *NSAIDs* Non-steroidal anti-inflammatory drugs, *MIDAS* Migraine disability assessment, *HIT-6* Headache impact test, *BAI* Beck anxiety inventory, *BDI-II* Beck depression inventory-second edition, *MAbs* Monoclonal antibodies

**Fig. 2:**
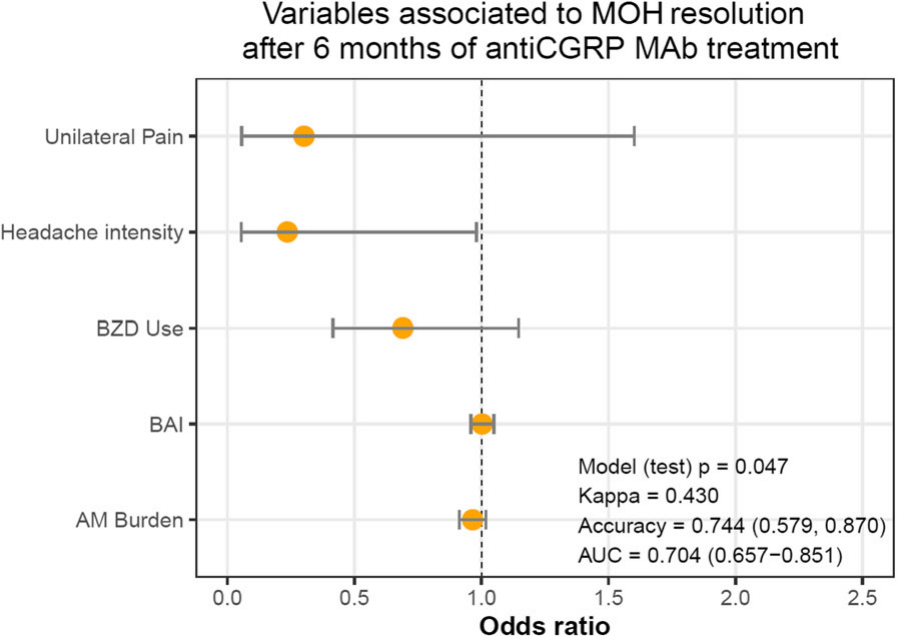
Odds Ratio (95% CI) estimated from the logistic regression analysis of the significant clinical predictors associated to MO resolution after 6 months of anti-CGRP MAbs treatment. Odds ratio (95% CI) estimated from the 10-fold CV of the stepwise logistic regression with selection criteria of minimizing the AIC for a variable to be eliminated from the selected model. The final logistic regression model, in the test set, presented an accuracy of 0.744 [0.579–0.870] and a Kappa value of 0.430 (*p* = 0.047). Only headache intensity remained as a statistically significantly independent predictor in the model (OR [95%]: 0.236[0.054–0.975]; *p* = 0.049). Unilateral Pain, BZD Use, BAI and AM burden were not statistically significant in the logistic regression analysis (If the 95% CI includes/crosses the value of 1, then there is insufficient evidence to conclude that these covariables are statistically significantly and independently associated to the outcome variable). MO: medication overuse, OR: odds-ratio; CI: confidence interval; BZD: benzodiazepines; AM: acute medication; BAI: Beck anxiety inventory; AUC: area under the curve

After the AIC stepwise selection of the significant variables, the final predictors introduced in the logistic regression multivariate analysis were: baseline MDM, headache pain intensity, unilateral pain (No/Yes), AM burden (pills/month), benzodiazepine use and BAI. We observed that only headache intensity remained as a statistically significant independent factor associated with MO resolution, meaning that having a lower baseline pain severity, was correlated with patients stopping medication overuse (OR[95%]: 0.236[0.054–0.975]; *p* = 0.049) (Fig. 2). In the test set, the model presented an accuracy of 0.744 [0.579–0.870], a Kappa value of 0.430 (*p* = 0.047) and AUC in the ROC of 0.704 [0.657–0.851].

## Discussion

In our real-life cohort of chronic migraine patients with MO, demographic data and baseline headache characteristics were similar to clinical trials [8–10], with the exception of longer disease duration and more refractoriness to preventive medication, in line with other real-world studies [4–7].

In this prospective study, after 6 months of treatment with anti-CGRP MAbs, we observed that:

First, ≥50% reduction in headache frequency for both MDM and HDM is similar regardless of the presence of MO at baseline. Our results are in line with other real-world multicenter CM cohorts for both erenumab (at 3 and 6 months) [4, 7] and galcanezumab (at 6 months) [6], all of which are higher than the reponse seen in clinical trials [13–15]. Moreover, patients with ongoing MO after 6 months do experience reduction in headache frequency similar to trials [8] and improve acute medication intake although not reaching a non-overuse behavior. This points out to the fact that anti-CGRP MAbs may be an effective preventive treatment for refractory migraine patients with MO. According to our results, they are also safe and well-tolerated.

Second, 60.6% of patients achieve remission of MO. This rate is comparable to the one observed by Ornello et al. [4] for erenumab (71.9%) but slightly lower than the one showed for galcanezumab in the GARLIT study (82.0%) [6]. The reason may be due to how MO remission was defined in our study as an absence of MO during 6-months of treatment and not only at one specific timepoint (6 months). However, our results are still better than the ones seen in clinical trials [9]; justifying the use in daily practice of anti-CGRP MAbs to facilitate acute medication discontinuation through their preventive effect.

Third, anti-CGRP MAbs help categorize MO into two profiles: 1) the MO-resolution group which represents patients whose overuse is directly related to migraine frequency, reducing days of acute medication intake by improving frequency with treatment; 2) the MO-ongoing group that includes patients where, despite a reduction in MDM, other factors such as higher pain intensity possibly prevent from achieving a non-overuse behavior. This may explain our finding of pain intensity as an independent factor of MO resolution. In this regard, pain severity may be seen as an expression of a worse basal disease phenotype leading to medication overuse rather than representing the consequence of it. More severe pain may lead to a greater analgesic intake even within a single headache day or use of benzodiazepine for pain relief. It is not surprising that these two factors were associated with ongoing MO, having these patients a significantly more severe headpain at baseline compared to the one the MO-resolution group had. However, these MO profiles and possible predictors of MO resolution should be further evaluated by long-term real-life studies and larger cohorts of treated patients with anti-CGRP MAbs.

Finally, neither the specific type of anti-CGRP MAbs (erenumab vs. galcanezumab) used, nor the presence of concomitant treatment with BTX-A could be considered as predictors of MO resolution. These findings have important implications in daily practice as they do not support (1) one anti-CGRP MAb being better than the other for MO patients and (2) using dual therapy with concomitant BTX-A instead of monotherapy.

The major limitation of this study is the presence of concomitant preventive treatments that make it difficult to exclusively evaluate the effect of anti-CGRP MAbs. However, this is a common bias in all real-life studies which we tried to reduce by keeping patients on stable doses of concomitant preventives.

Our real-life study therefore supports the use of anti-CGRP drugs in patients with chronic migraine and MO with similar results as compared to those who do not overuse. Moreover, the preventive effect of anti-CGRP monoclonal antibodies facilitates acute medication discontinuation and achieves reduction in headache frequency and acute medication use days regardless of patients stopping overuse.

## Supplementary Information

**Additional file 1.**

## Data Availability

All data are available and any anonymized data will be shared by request from any qualified investigator.

## Abbreviations

MO: Medication overuse
CM: Chronic migraine
CGRP: Calcitonin gene-related peptide
MAbs: Monoclonal antibodies
BTX-A: onabotulinumtoxinA
MDM: Migraine days/month
HDM: Headache days/month
AM: Acute medication
NSAIDs: Non-steroidal anti-inflammatory drugs
MO-R: MO resolution group
MO-O: MO-ongoing group
AUC: Area under the curve
BAI: Beck Anxiety Inventory scale
