# Supplementary material for: Anti-CGRP monoclonal antibodies in chronic migraine with medication overuse: real-life effectiveness and predictors of response at 6 months
Source: J Headache Pain. 2021 Oct 7;22(1):120. doi: 10.1186/s10194-021-01328-1 (PMC8499507; doi:10.1186/s10194-021-01328-1)
Supplement: Supplementary file 1 — Additional file 1. [file 10194_2021_1328_MOESM1_ESM.docx]

**Supplementary Table 1. Baseline characteristics and treatment response after 6 monthly administrations of anti-CGRP MAbs in patients with and without medication overuse**

| ***Without MO***  *(n=40)* | ***With MO***  *(n=99)* | *P value* |  |
| --- | --- | --- | --- |
| **Demographics** | | | |
| Age, mean (SD) years | 45.2 (10.4) | 47.7 (9.09 | 0.158**^§^** |
| Gender (female), n (%) | 32 (80.0) | 81 (81.8) | 0.803**^†^** |
| **Disease characteristics** | | | |
| Duration of migraine disease, mean (SD) y | 24.3 (11.9) | 26.4 (13.2) | 0.373**^§^** |
| Chronification time, mean (SD) y | 12.0 (6.4) | 12.3 (7.6) | 0.793**^§^** |
| **Aura, n (%)** | 18 (45.0) | 23 (23.2) | **0.011^†^** |
| Allodynia, n (%) | 22 (55.0) | 49 (49.5) | 0.557**^†^** |
| Unilateral pain side, n (%) | 33 (82.5) | 75 (75.8) | 0.387**^†^** |
| Pain quality, n (%)  Oppressive  Throbbing | 14 (35.0)  23 (57.5) | 48 (48.5)  67 (67.6) | 0.148^†^  0.256^†^ |
| HDM, mean (SD), d/mo  MDM, mean (SD), d/mo  Headache pain intensity, mean (SD), 0-3 score | 23.2 (5.6)  16.7 (6.4)  1.58 (0.50) | 23.3 (5.8)  17.8 (6.6)  1.70 (0.47) | 0.894^§^  0.396^§^  0.168^§^ |
| **Preventive treatment** | | | |
| Anti-CGRP Treatment, n (%)  Erenumab 140mg  Galcanezumab 120mg (240mg initial dose) | 27 (67.5)  13 (32.5) | 69 (69.7)  30 (30.3) | 0.800**^†^** |
| Prior preventive classes failures, n (%)  3 Classes  4 Classes  ≥5 Classes | 9 (22.5)  10 (25.0)  21 (52.5) | 8 (8.1)  39 (39.4)  52 (52.5) | 0.284**^†^** |
| Prior BTX-A efficacy^*^, n (%)  Partial  Failure | 17/33 (51.5)  16/33 (48.5) | 54/93 (58.1)  39/93 (41.9) | 0.515**^†^** |
| Concomitant preventive treatment, n (%)  **Oral concomitant medication, n (%)**  BTX-A concomitant medication, n (%) | 31 (77.5)  31 (77.5)  13 (32.5) | 65 (65.7)  57 (57.6)  38 (38.4) | 0.171^†^  **0.027^†^**  0.515^†^ |
| **Acute medication** | | | |
| **Acute medication frequency, mean (SD), d/mo**  **NSAIDs frequency, mean (SD), d/mo**  **Triptans frequency, mean (SD), d/mo**  BZD frequency, mean (SD), d/mo | 6.8 (4.4)  3.3 (2.9)  3.5 (3.2)  1.3 (0.3) | 19.2 (7.0)  9.1 (8.5)  12.9 (7.7)  3.5 (0.9) | **<0.0001^§^**  **<0.0001^§^**  **<0.0001^§^**  0.108 |
| **Acute medication burden, mean (SD), p/mo** | 8.3 (5.4) | 26.9 (11.7) | **<0.0001^§^** |
| **Disease impact, disability and burden** | | | |
| **Disability (MIDAS), median [IQR]** | 54.5 [66.3] | 85.0 [67.0] | **0.005^‡^** |
| Headache-related impact (HIT-6), mean (SD) | 72.1 (30.1) | 68.4 (15.1) | 0.473^§^ |
| Anxiety (BAI), median [IQR] | 20.0 [25.3] | 19.0 [20.0] | 0.717^‡^ |
| Depression (BDI-II), median [IQR] | 14.5 [17.8] | 11.0 [17.0] | 0.589^‡^ |
| **Treatment response rate at 6 months** | | | |
| HDM 50% RR, n (%)  MDM 50% RR, n (%) | 17 (42.5)  23 (57.5) | 53 (53.5)  63 (63.6) | 0.239^‡^  0.500^‡^ |

**Bold** font indicates statistically significant variables.

**^*^**126/139 patients were previously treated with BTX-A

**^§^**Significance assessed with independent *t*-test

**^†^**Significance assessed with Fisher’s exact test or linear trend chi-square test (preventive classes failures)

**^‡^**Significance assessed with Mann-Whitney U test

MO: medication overuse; SD: standard deviation; IQR: interquartile range; y: years; d/mo: days per month; p/mo: pills per month; HDM: headache days/month; MDM: migraine days/month; BTX-A: OnabotulinumtoxinA; NSAIDs: non-steroidal anti-inflammatory drugs; MIDAS: migraine disability assessment; HIT-6: headache impact test; BAI: Beck anxiety inventory; BDI-II: Beck depression inventory-second edition. MAbs: monoclonal antibodies
